# HYOU1 facilitates proliferation, invasion and glycolysis of papillary thyroid cancer via stabilizing LDHB mRNA

**DOI:** 10.1111/jcmm.16453

**Published:** 2021-03-31

**Authors:** Jia‐Mei Wang, Jing‐Yi Jiang, Da‐Lin Zhang, Xin Du, Tong Wu, Zhen‐Xian Du

**Affiliations:** ^1^ Department of Endocrinology & Metabolism The 1st Affiliated Hospital China Medical University Shenyang China; ^2^ Clinical medical laboratory The 1st Affiliated Hospital China Medical University Shenyang China; ^3^ Department of Biochemistry & Molecular Biology China Medical University Shenyang China; ^4^ Department of Thyroid Surgery The 1st Affiliated Hospital China Medical University Shenyang China

**Keywords:** glycolysis, HYOU1, LDHB, papillary thyroid cancer

## Abstract

HYOU1 is upregulated in many kinds of cancer cells, and its high expression is associated with tumour invasiveness and poor prognosis. However, the role of HYOU1 in papillary thyroid cancer (PTC) development and progression remains to be elucidated. Here, we reported that HYOU1 was highly expressed in human PTC and associated with poor prognosis. HYOU1 silencing suppressed the proliferation, migration and invasion of PTC cells. Mechanistic analyses showed that HYOU1 silencing promoted oxidative phosphorylation while inhibited aerobic glycolysis via downregulating LDHB at the posttranscriptional level. We further confirmed that the 3'UTR of LDHB mRNA is the indirect target of HYOU1 silencing and HYOU1 silencing increased miR‐375‐3p levels. While LDHB overexpression significantly suppressed the inhibitory effects of HYOU1 silencing on aerobic glycolysis, proliferation, migration and invasion in PTC cells. Taken together, our findings suggest that HYOU1 promotes glycolysis and malignant progression in PTC cells via upregulating LDHB expression, providing a potential target for developing novel anticancer agents.

## 1 INTRODUCTION

Thyroid cancer is the most prevalent endocrine malignancy and there has been an increase in the incidence of thyroid cancers all over the world.^1^ Papillary thyroid cancer (PTC) is the main form of thyroid cancer and accounts for between 80% and 85% of all thyroid malignancies.^2^ The majority of PTCs generally have a favourable prognosis.^3^ However, a subset of PTCs may exhibit aggressive behaviour,^4^, ^5^ including rapid growth, local recurrence and distant metastasis. Such PTCs with aggressive behaviour usually have a poorer outcome. Therefore, there is a need for further in‐depth investigations of the molecular mechanisms underlying PTC invasive behaviour.

Hypoxia up‐regulated 1 (HYOU1), also known as ORP150 or Grp170 (glycosylated form of ORP150), belongs to the heat shock protein 70 family. HYOU1 plays an important role in protein folding in the endoplasmic reticulum (ER).^6^ Previous data show that HYOU1 exerts an important cytoprotective role under stressful conditions, such as hypoxia and glucose deprivation.^7^, ^8^ Recent evidence suggests that HYOU1 is upregulated in a series of tumours, such as breast tumours, nasopharyngeal carcinoma, epithelial ovarian cancer and Kaposi's sarcoma.^9^, ^10^, ^11^, ^12^ Furthermore, increased HYOU1 expression is associated with tumour invasiveness and poor prognosis. However, potential mechanism of HYOU1 in the development and progression of tumours remains largely unclear.

In this study, we observed that HYOU1 was highly expressed in PTC. Our data demonstrated that silencing of HYOU1 inhibited proliferation, migration and invasion of PTC cells. Furthermore, we validated that HYOU1 facilitated glycolysis via upregulation of LDHB to promote aggressive progression of PTC cells. We further elucidated that HYOU1 indirectly increased the expression of LDHB at the post‐transcriptional level. These findings revealed a new insight into the involvement of HYOU1 in reprogramming of glucose metabolism, widening the knowledge of the molecular mechanisms underlying PTC aggressive behaviour.

## 2 MATERIALS AND METHODS

### 2.1 Cell culture

Human PTC cell lines, IHH4, TPC1, K1 and BCPAP were obtained from ATCC and cultured in Dulbecco's Modified Eagle's Medium (DMEM) supplemented with 10% foetal bovine serum (FBS) at 37°C in a humidified atmosphere with 5% CO_2_.

### 2.2 Patients and tissue specimens

A total of 115 pairs of PTC tissues and corresponding noncancerous thyroid tissues were collected from patients who underwent surgical resection at the first affiliated hospital of China Medical University between June 2017 and May 2019. Patients with a history of other malignancies or those who received preoperative radiotherapy or chemotherapy were excluded from this study. Written informed consent was signed by each subject, and this study was approved by the Ethics Committee of China Medical University and abided by the Declaration of Helsinki. All specimens were frozen in liquid nitrogen immediately and stored at −80℃ until use.

### 2.3 Western blotting

The proteins in PTC cells or frozen PTC and adjacent tissues were extracted using the Total Protein Extraction Kit (Thermo Fisher). The protein extracts (20 μg) for each sample were separated by 10% SDS‐PAGE and transferred electrophoretically to polyvinylidene fluoride membrane (Millipore). After being blocked in 5% skimmed milk for one hour, the membranes were incubated overnight at 4°C with primary antibodies recognizing HYOU1, HK2, PKM1/2 (Cell Signaling Technology), LDHA, LDHB (Abcam), and β‐Actin, GAPDH (Santa Cruz). After incubation with secondary antibodies (ZSGB‐BIO), the protein bands were visualized with an imaging system (Tanon‐5800, Tanon Science & Technology Co., Ltd). Gray values of HYOU1/β‐Actin were analysed by Image J Statistical analysis.

### 2.4 Cell proliferation assay

Cells were seeded in 12‐well plates in triplicates at 2 × 10^5^ cells per well and cultured in complete medium for 24, 48 and 72 hours. At the indicated time points, cells were trypsinized and counted using a haemocytometer.

### 2.5 5‐ethynyl‐2′‐deoxyuridine (EdU) Incorporation Assay

EdU incorporation assay was performed with the Click‐iT EdU imaging kit (Thermo Fisher Scientific), according to the supplier's instruction. Briefly, 500 μL 5 × 10^4^ cells per well were seeded in 24‐well plates and incubated overnight. Then, 250 μL fresh media containing 20 μM of EdU replaced half of old media. Then the cells were cultured for another 8 hours. Subsequently, the cells were fixed using 3.7% formaldehyde for 15 minutes and permeated with 0.5% Triton X‐100 for 20 minutes. After rinsing with 3% bovine serum albumin, 500 μL of 1 ×Click‐iT reaction cocktail was added and incubated for 30 minutes at room temperature, avoiding from light. Then 5 μg/mL Hoechst 33342 (Thermo Fisher Scientific) was added to stain cell nuclei for 30 minutes, protected from light. The results were determined by fluorescence microscopy (Olympus CKX‐31). All assays were conducted independently three times in triplicate.

### 2.6 Cell migration and invasion assay

The transwell system (Corning Incorporated Life Sciences) was adopted for cell migration and invasion assays as described previously. In brief, cells in serum‐free DMEM were seeded onto the upper chambers. The lower chambers were filled with DMEM containing 10% FBS. After incubation for 24 hours, cells on the interior surfaces of upper chambers were fixed by methanol, then stained using crystal violet. The invasion assay shared the same procedures, except that the filter inserts were precoated with Matrigel. The migrated or invaded cells were counted in ten representative microscopic fields and photographed.

### 2.7 Measurement of oxygen consumption rate (OCR) and extracellular acidification (ECAR)

The extracellular acidification rate (ECAR) and cellular oxygen consumption rate (OCR) were determined using the Seahorse XF24 Extracellular Flux Analyser (Seahorse Bioscience) according to the manufacturer's protocols. Briefly, 2 × 10^4 ^cancer cells were seeded into a 24‐well plate followed by overnight incubation. After the cells were washed with Seahorse assay medium, for ECAR, glucose, oligomycin, and the 2‐DG were automatically and sequentially injected at indicated time points. For OCR, oligomycin, FCCP (p‐trifluoromethoxy carbonyl cyanide phenylhydrazone), and the mitochondrial complex III inhibitor antimycin A were sequentially injected.

### 2.8 Determination of the extracellular lactate and glucose

Glucose consumption and L‐lactate production were examined using Lactate Colorimetric Assay Kits (K627, Biovision) and Glucose Uptake Colorimetric Assay Kits (K686, Biovision) according to the manufacturer's instructions.

### 2.9 Label and capture of nascent RNA

To detect newly synthesized RNA, a Click‐iT Nascent RNA Capture kit (Thermo Fisher Scientific) was used as described by the manufacturer. In brief, cells were incubated with 5‐ethynyl uridine (EU) for 4 hours. Total RNA was obtained using TRIzol reagent (Invitrogen), and the EU‐nascent RNA is efficiently captured on magnetic beads for subsequent cDNA synthesis and qPCR.

### 2.10 Luciferase assay

The full‐length 5′ untranslated region (5′UTR), coding region (CR), and 3′ UTR of human LDHB mRNA was cloned into pGL4 basic vector. The construct containing mutant for the seed sequence of miR‐375‐3p was obtained through PCR‐based site‐directed mutagenesis kit (Agilent Technologies). The transfection was performed with Lipofectamine 2000 according to the manufacturer's instructions. The cells were harvested for firefly/Renilla luciferase assays 48 hours after transfection using the Dual‐Luciferase Reporter Assay System (Promega). All experiments were done in triplicate and were independently performed at least three times.

### 2.11 Statistical analysis

Statistical analysis was performed using SPSS 16.0 software (SPSS). All results were presented as the mean ± standard deviation. All tests were two‐tailed, and a *P*‐value <0.05 was considered statistically significant.

## 3 RESULTS

### 3.1 HYOU1 is highly expressed in papillary thyroid cancer and related to poor prognosis

**FIGURE 1:**
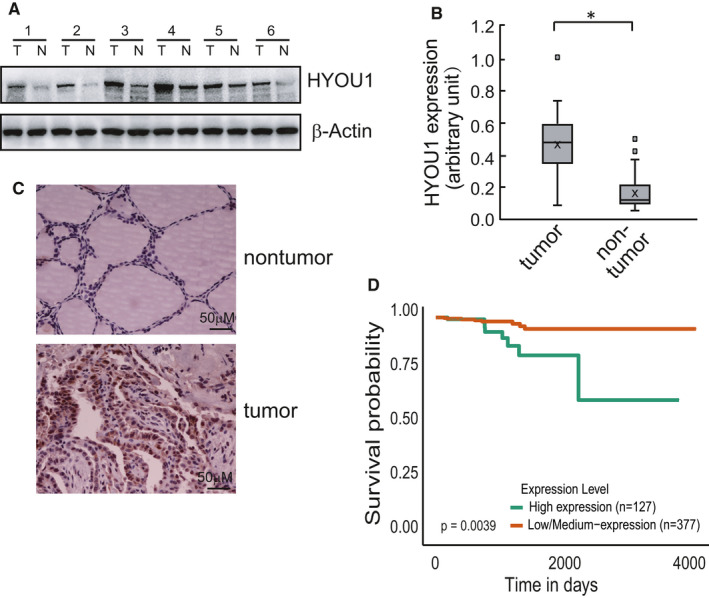
Higher HYOU1 gene expression is associated with poor prognosis in PTC. A, Representative immunoblot images of HYOU1 are shown. N: adjacent normal tissues; T: PTC tissues. B, Densitometry analysis of immunoblot results, the expression of HYOU1 was qualified compared with the expression of β‐Actin. The differences of gray values of HYOU1/β‐Actin between PTC tissues and adjacent normal tissues was analysed by paired *t* test. C, Representative images of immunostaining of HYOU1 in PTC tissues (the upper image) and normal tissues (the lower image). D, Kaplan‐Meier analysis of relapse‐free survival in publicly available PTC datasets. Patients were divided according to HYOU1 expression as indicated

HYOU1 expression was firstly detected in 72 pairs of PTC tissues and their matched normal thyroid tissues via western blot, and the results demonstrated that HYOU1 was highly expressed in PTC tissues compared with adjacent normal thyroid tissues (Figure 1A‐B). Elevated expression of HYOU1 in PTC issues was further validated by immunohistochemistry analysis (Figure 1C). The association between HYOU1 expression and prognosis of the patients with thyroid cancer of TCGA database was analysed using online web resource UALCAN (http://ualcan.path.uab.edu/) and high expression of HYOU1 was demonstrated to be associated with poor prognosis of thyroid cancer (Figure 1D). These findings preliminarily indicated that HYOU1 may play an oncogenic role in thyroid cancer.

### 3.2 HYOU1 silencing represses proliferation, migration and invasion of papillary thyroid cancer cells

**FIGURE 2:**
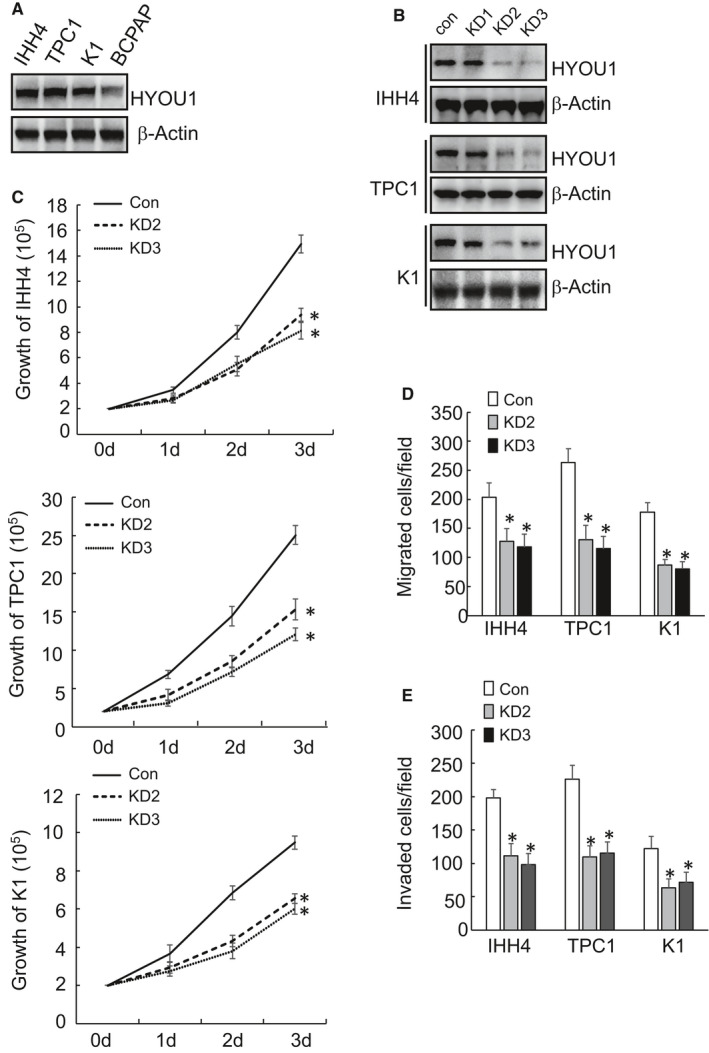
Silence of HYOU1 inhibit proliferation, migration and invasion of papillary thyroid cancer cells. A, Western blot analyses showed that HYOU1was higher in IHH4, TPC1 and K1 cell lines and lower in BCPAP cell line. B, siRNA for HYOU1 or control nonspecific siRNA were transfected individually into IHH4, TPC1 and K1 cells. Western blot was performed to determine the effects of HYOU1 silencing. C, 2 × 10^5^ of control or HYOU1‐silencing PTC cells were seeded in 12‐well plates. Cell proliferation was examined with cell counting. D, Cell migration was determined in IHH4, TPC1 and K1 cells using Corning Transwell system. The migrated cells were counted under a microscope. E, Cell invasion was examined by Corning Matrigel invasion assay

To explore the molecular function of HYOU1, we investigated the expression of HYOU1 in multiple thyroid cancer cell lines, including IHH4, TPC1, K1 and BCPAP. High level of expression of HYOU1 was found in IHH4, TPC1, K1 cells, while relatively lower HYOU1 expression was detected in BCPAP cells (Figure 2A, Figure S1A). Expression of HYOU1 was then silenced in IHH4, TPC1 and K1 cells by transfecting HYOU1‐targeting shRNA to identify the potential involvement of HYOU1 in PTCs. HYOU1 silencing was verified by western blot (Figure 2B, Figure S1B). HYOU1 silencing significantly inhibited the proliferation of IHH4, TPC1 and K1 cells (Figure 2C). Transwell assays demonstrated that HYOU1 inhibition also significantly restrained migration (Figure 2D) and invasion (Figure 2E) of IHH4, TPC1 and K1 cells. The above results indicated that HYOU1 silencing could impede malignant behavior of PTC cells.

### 3.3 Silencing of HYOU1 inhibited aerobic glycolysis

**FIGURE 3:**
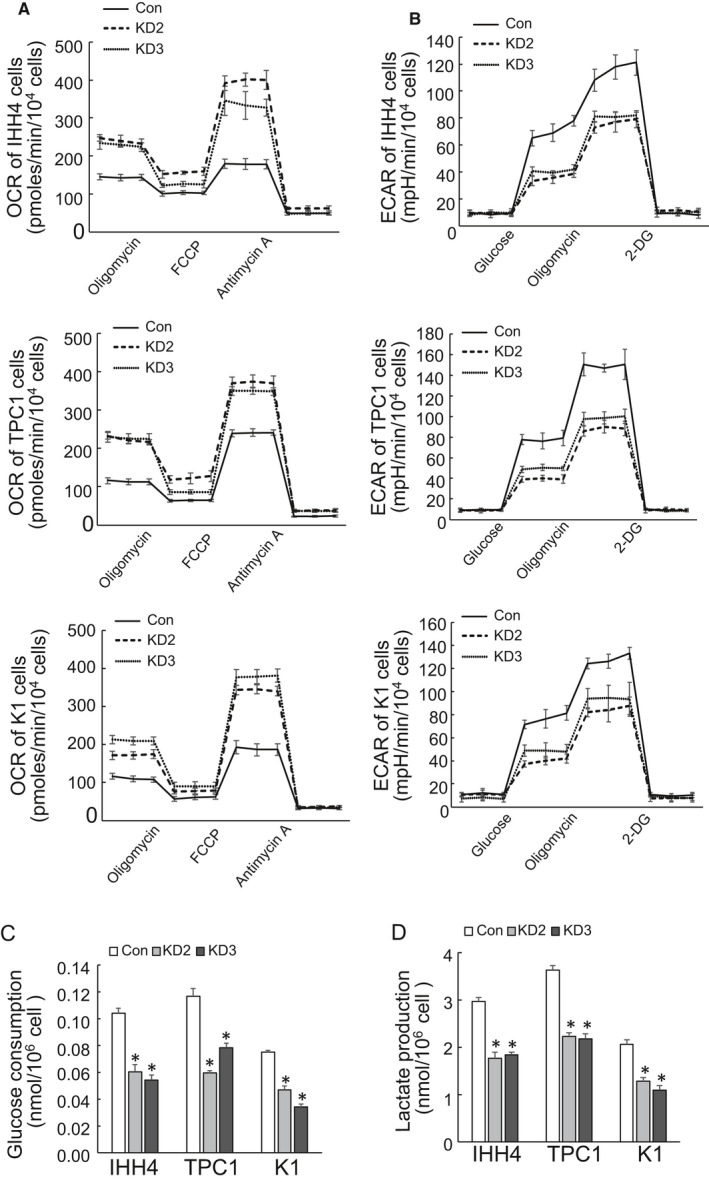
Silence of HYOU1 inhibits aerobic glycolysis. A, OCR was measured using seahorse instrument in control or HYOU1‐silenced IHH4, TPC1 and K1 cells. B, ECAR was analysed using seahorse instrument in control or HYOU1‐silenced IHH4, TPC1 and K1 cells. C, Glucose consumption was determined using colorimetric method in control or HYOU1‐silenced IHH4, TPC1 and K1 cells. D, Extracellular lactate production was analysed using colorimetric method in control or HYOU1‐silenced IHH4, TPC1 and K1 cells. All values displayed are mean ± SD. *P < 0.01, versus corresponding Con

To investigate a potential impact of HYOU1 on metabolic regulation of PTC cells, mitochondrial function was monitored by real‐time measurement of changes in the oxygen consumption rate (OCR) after sequential treatment with oligomycin, the cyanide p‐trifluoromethoxyphenyl‐hydrazone (FCCP), or antimycin A. HYOU1 silencing resulted in a significant rise of OCR in IHH4, TPC1 and K1 cells (Figure 3A). The real‐time extracellular acidification rate (ECAR), a measure of glycolysis, was also assessed after serial addition of glucose, oligomycin, and 2‐deoxyglucose. In contrast, there was a significant drop in the level of ECAR when HYOU1 was silenced in IHH4, TPC1 and K1 cells, indicating a decrease of glycolysis (Figure 3B). The decline of glycolysis was further confirmed by the lower level of secreted lactic acid, the major by‐product of glycolysis (Figure 3D). Extracellular glucose was also detected, and a significant decrease in glucose consumption was observed in IHH4, TPC1 and K1 cells with HYOU1 silencing (Figure 3C). Taken together, the above data suggested that HYOU1 silencing suppressed glycolysis, but promoted mitochondrial respiration.

### 3.4 HYOU1 silencing suppressed proliferation, migration and invasion of IHH4 and K1 cells by downregulation of LDHB

**FIGURE 4:**
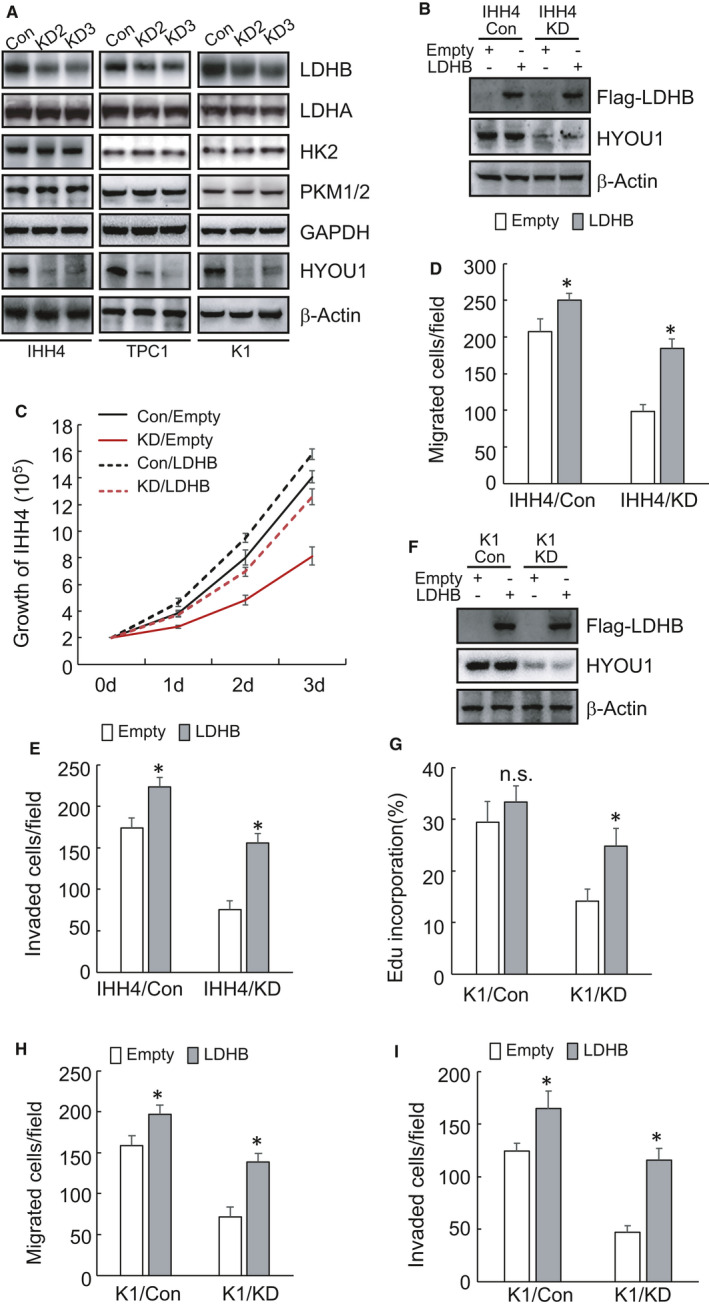
Silence of HYOU1 downregulates LDHB to suppress the proliferation, migration and invasion of IHH4 and K1 cells. A, The expression of genes associated with glycolysis was detected in control or HYOU1‐silenced IHH4, TPC1 and K1 cells by western blot. B, F, Control or HYOU1‐silenced IHH4 and K1 cells were infected with lentivirus containing empty or LDHB construct. LDHB overexpression was confirmed using western blot. C, The proliferation of control or LDHB‐ overexpression IHH4 cells was measured by cell counting. D, E, The migration and invasion of control or LDHB‐overexpression IHH4 cells was detected via Corning Transwell assay separately. G, De novo DNA synthesis was analysed using Edu incorporation in control or LDHB‐overexpression K1 cells. H, I, The migration and invasion of control or LDHB‐overexpression K1 cells was detected via Corning Transwell assay separately. All values displayed are mean ± SD. *P < 0.01, versus corresponding Con

To explore the potential mechanism of HYOU1 involved in glycolysis, we analysed the expression of a series of genes related to glucose metabolism, including LDHA, LDHB, HK2, PKM1/2, and GAPDH. Western blot demonstrated that the expression level of LDHB was significantly decreased by HYOU1 silencing, while the expression level of LDHA, HK2, PKM1/2 and GAPDH did not change significantly (Figure 4A, Figure S1C). To investigate whether HYOU1 regulated glycolysis through LDHB, LDHB was overexpressed in IHH4 cells with HYOU1 silencing (Figure 4B, Figure S1D). Cell counting results demonstrated that LDHB overexpression compromised the suppression of proliferation induced by inhibition of HYOU1 in IHH4 cells (Figure 4C). Transwell assay data showed that LDHB overexpression diminished the inhibition of migration and invasion by HYOU1 inhibition in IHH4 cells (Figure 4D,E). Similar results were obtained in K1 PTC cells (Figure 4F‐I, Figure S1E).

### 3.5 HYOU1 promotes glycolysis in papillary thyroid cancer cells via upregulation of LDHB

**FIGURE 5:**
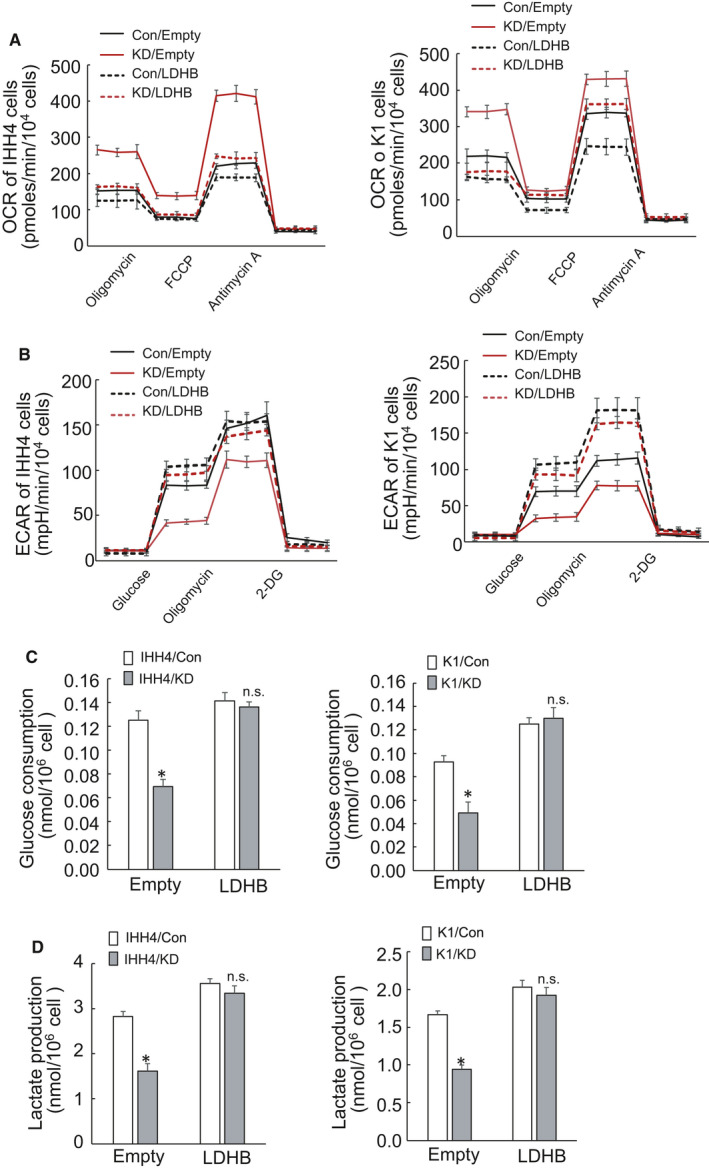
LDHB overexpression reversed fully the inhibition of glycolysis by HYOU1 silencing. A, OCR was measured using seahorse instrument in control or LDHB‐overexpression IHH4 and K1 cells with control or HYOU1‐silencing. B, ECAR was analysed using seahorse instrument in the same cells as A. C, Glucose consumption was determined using colorimetric method in the same cells as A. D, Extracellular lactate production was analysed using colorimetric method in the same cells as A. All values displayed are mean ± SD. *P < 0.01, versus corresponding Con

Real‐time measurement of the OCR demonstrated that LDHB overexpression significantly decreased the OCR in both HYOU1 silenced and control IHH4 and K1 cells, particularly sharply in IHH4 and K1 cells with HYOU1 silencing (Figure 5A). While the ECAR was increased significantly by LDHB overexpression in both HYOU1 silenced and control IHH4 and K1 cells (Figure 5A). Extracellular glucose analysis demonstrated that LDHB overexpression reversed the decrease of glucose consumption caused by silencing of HYOU1 (Figure 5C). Meanwhile, the decrease of extracellular lactate secretion induced by HYOU1 silencing was reversed by ectopic overexpression of LDHB (Figure 5D). These data suggested that HYOU1 facilitates glycolysis in PTC cells through LDHB upregulation in PTC cells.

### 3.6 HYOU1 regulates the stability of LDHB mRNA via its 3′UTR

**FIGURE 6:**
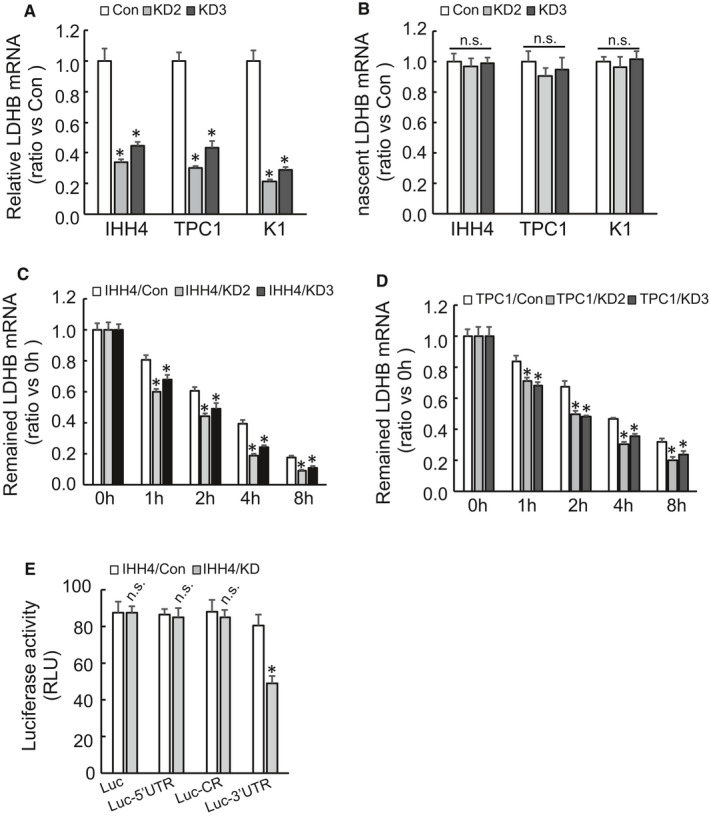
HYOU1 regulates stability of LDHB mRNA via its 3′UTR. A, LDHB mRNA was analysed using RT‐PCR in control or HYOU1‐silenced IHH4, TPC1 and K1 cells. B, New synthetic RNA was labeled and isolated, and newly synthesized LDHB mRNA was analysed using RT‐PCR in control or HYOU1‐silenced IHH4, TPC1 and K1 cells. C, D, RNA synthesis was blocked by addition of Actinomycin D, and LDHB mRNA was determined using RT‐PCR in control or HYOU1‐silenced IHH4 and TPC1 cells. E, Control or HYOU1‐silenced IHH4 cells were transfected with the indicated recombined firefly luciferase reporter vector and a Renilla luciferase reporter vector. Luciferase activity was measured 48 hours after transfection, and firefly luciferase activity was normalized to Renilla luciferase activity. *P < 0.01. Error bars indicate means ± SD

To investigate the possible mechanism by which HYOU1 upregulates LDHB, the level of LDHB mRNA was detected by Reverse transcription‐quantitative PCR (RT‐qPCR) after silencing of HYOU1. We observed that HYOU1 silencing significantly decreased the abundance of LDHB mRNA in IHH4, TPC1, and K1 cells (Figure 6A). However, new LDHB mRNA synthesis determined by a Click‐iT nascent RNA capture systems demonstrated that HYOU1 silencing did not alter the novel synthesis of LDHB mRNA in IHH4, TPC1, and K1 cells (Figure 6B). Then we measured the stability of the LDHB mRNA in IHH4 and TPC1 cells after de novo RNA synthesis was blocked by Actinomycin D. Our results suggested that HYOU1 silencing promoted degradation of the LDHB mRNA in IHH4 (Figure 6C) and TPC1 (Figure 6D) cells. To identify the regulatory region in LDHB mRNA by HYOU1, the 5′UTR, CR, and 3′ UTR of LDHB mRNA was inserted just after the stop codon of the luciferase reporter gene respectively. Compared with control cells, IHH4 cells with HYOU1 silencing demonstrated a significant decrease in the luciferase activity of the construct containing 3′UTR of LDHB mRNA (Figure 6E). While insertion of 5′UTR or CR of LDHB mRNA showed no significant changes in luciferase activity in control and HYOU1 silenced cells (Figure 4E). These data indicate that HYOU1 regulates the stability of LDHB1 mRNA via its 3′UTR in PTC cells.

### 3.7 Decreased stability of LDHB mRNA caused by HYOU1 silencing is related to the increase of miR‐375‐3p

**FIGURE 7:**
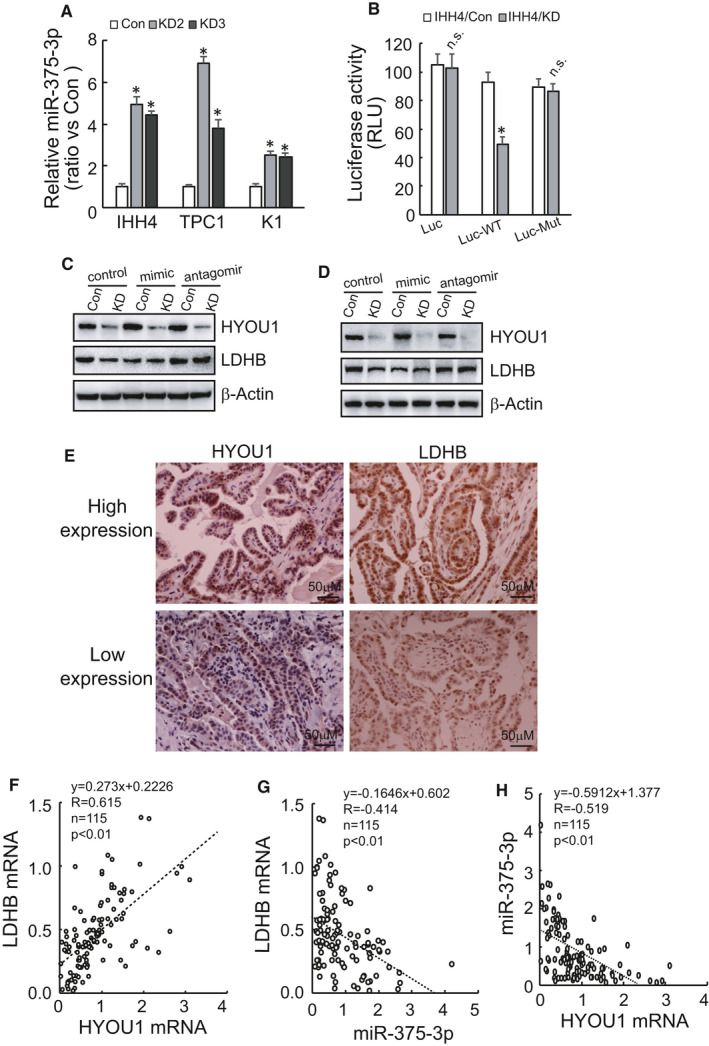
Decreased stability of LDHB mRNA caused by HYOU1 silencing is related to the increase of miR‐375‐3p. A, The expression of miR‐375 was analysed using RT‐PCR in control or HYOU1‐silenced IHH4, TPC1 and K1 cells. B, Luciferase activity was measured in control or HYOU1‐silenced IHH4 cells transfected with luciferase reporter vector bearing wild‐type or mutant 3′UTR of LDHB mRNA. C, D, Western blot analysis of LDHB in control or HYOU1‐silenced IHH4 or K1 cells transfected miR‐375 mimics and antagomir. E, Representative images of immunostaining of HYOU1 and LDHB in PTC tissues. F‐H, HYOU1 mRNA, LDHB mRNA and miR‐375 levels were measured in 115 PTC tissues using RT‐qPCR. The correlation among them was assessed by Spearman's rank correlation

As HYOU1 is not an RNA‐binding protein, we hypothesize that HYOU1 may indirectly regulate the stability of LDHB mRNA through some mediator. As is well known, miRNAs can hybridize with target mRNAs at the 3′UTR to regulate gene expression at the posttranscriptional level. GeneChip analysis screened that 7 and 5 miRNAs were significantly upregulated and downregulated by HYOU1 silencing in IHH4 cells, respectively (Data S1). Upregulation of miR‐375‐3p in IHH4 with HYOU1 silencing attracted our attention, for the following reasons. Firstly, online data from Starbase indicated the negative correlation between HYOU1 and miR‐375‐3p expression (coefficient‐R = −0.380, *P* = 5.95e‐19) in thyroid cancer. Secondly, two online predicative tools (TargetScan and miRDB) predicted that LDHB mRNA is the target of miR‐375‐3p, while no potential binding sites on 3′UTR of HYOU1 mRNA. Thirdly, bioinformatics analysis using Starbase data demonstrated significant negative correlation between LDHB mRNA and miR‐375‐3p expression in many kinds of cancer, including thyroid cancer (Table 1). Besides, it has been reported that miR‐375‐3p regulates the expression of LDHB.^13^ RT‐qPCR results confirmed that HYOU1 silencing led to a significant increase of miR‐375‐3p in IHH4, TPC1, and K1 cells (Figure 7A). Luminescence intensity was significantly reduced by transfection of wild‐type of the LDHB 3'UTR in IHH4 cells with HYOU1 silencing, while the LDHB 3'UTR with mutation in miR‐375‐3p target sites blocked the decrease in luminescence (Figure 7B). Subsequently, we confirmed that transfection of miR‐375‐3p mimics significantly decreased LDHB expression in control IHH4 (Figure 7C, Figure S1F) and K1 (Figure 7D, Figure S1G) cells, while had no obvious effect on LDHB protein expression in IHH4 (Figure 7C, Figure S1F) and K1 (Figure 7D, Figure S1G) cells with HYOU1 silencing. These data indicated that HYOU1 silencing alone might result in the maximum effect of miR‐375‐3p on LHDB expression, thereby excessive miR‐375‐3p exerted no obvious effect. On the contrary, miR‐375 antagomir significantly increased LDHB expression in IHH4 (Figure 7C, Figure S1F) and K1 (Figure 7D, Figure S1G) cells with HYOU1 silencing, while demonstrated no significant difference in control IHH4 (Figure 7C) and K1 (Figure 7D) cells. We also observed that there was an inverse correlation between HYOU1 and LDHB by immunohistochemical staining (Figure 7E). Also, the level of HYOU1 mRNA was significantly positively related to that of LDHB mRNA (Figure 7F) in 115 cases of PTC tissues, consistent with bioinformatics analysis obtained from Cancer Genome Atlas (Table 2). There was a significantly negative correlation between the level of LDHB mRNA and that of miR‐375(Figure 7G) in the same 115 cases of PTC tissues. In addition, the level of HYOU1 mRNA was inversely correlated to that of miR‐375 (Figure 7H). These data indicate a possibility that HYOU1 might upregulate the expression of LDHB through indirectly downregulating miR‐375 in PTCs.

**TABLE 1:** Pan‐Cancer co‐expression analysis of hsa‐miR‐375 and LDHB

Cancer	Sample number	coefficient‐R	*P*‐value
Bladder urothelial carcinoma	408	−0.116	1.90E‐02
Breast invasive carcinoma	1085	−0.348	3.64E‐32
Cholangiocarcinoma	36	−0.415	1.19E‐02
Esophageal carcinoma	162	−0.400	1.36E‐07
Brain lower grade glioma	525	−0.251	5.13E‐09
Pheochromocytoma and paraganglioma	183	−0.466	2.88E‐11
Prostate adenocarcinoma	495	−0.418	2.42E‐22
Thyroid carcinoma	509	−0.398	8.62E‐21
Uterine corpus endometrial carcinoma	538	−0.219	2.91E‐07

Note

Data Source: starBase v3.0 project.

**TABLE 2:** Pan‐Cancer co‐expression analysis of HYOU1 and LDHB

Cancer	Sample number	Coefficient‐R	*P*‐value
Breast invasive carcinoma	1104	0.274	1.61E‐20
Cholangiocarcinoma	36	−0.355	3.34E‐02
Colon adenocarcinoma	471	0.207	5.97E‐06
Head and neck squamous cell carcinoma	502	0.147	9.63E‐04
Kidney chromophobe	65	0.457	1.29E‐04
Kidney renal clear cell carcinoma	535	0.275	9.72E‐11
Brain low grade glioma	529	−0.159	2.42E‐04
lung adenocarcinoma	526	0.144	8.92E‐04
Pheochromocytoma and paraganglioma	183	0.158	3.31E‐02
Rectum adenocarcinoma	167	0.230	2.80E‐03
Skin cutaneous melanoma	471	0.155	7.20E‐04
Testicular germ cell tumors	156	0.457	1.99E‐09
Thyroid carcinoma	510	0.568	7.32E‐45
Uterine corpus endometrial carcinoma	548	0.307	1.87E‐13

Note

Data Source: starBase v3.0 project.

## 4 DISCUSSION

The incidence of PTC has been rapidly increasing over the past few decades.^14^ Although the prognosis of patients with PTC is generally favorable,^15^ local recurrence and distant metastasis are potentially life‐threatening that lead to an unfavorable outcome.^5^, ^16^ Many factors increase the risk of recurrence from PTC, including follicular variant of PTC, older age, lymph node metastasis and genetic mutations.^2^, ^16^, ^17^, ^18^ Tumor metastasis is a multistep and extremely complex process for cancer cells, including detaching from the primary tumor, then disseminating through the vascular or lymphatic channels, finally colonizing at the secondary site. Many signaling pathways and molecules are involved in regulating the above processes.^19^ Therefore, elucidating the molecular mechanisms underlying the recurrence and metastasis of PTC may contribute to intervene in malignant progression and develop targeted treatment of PTCs.

The present study demonstrated that the expression of HYOU1 was significantly increased in PTC tissues, and data from the Cancer Genome Atlas indicated close association between high expression of HYOU1 and poor prognosis. Increased expression of HYOU1 was confirmed in a series of PTC cell lines. Our results also showed that silencing of HYOU1 could suppress proliferation, migration and invasion in PTC cell lines, indicating that HYOU1 may play oncogenic function to promote aggressive progression of PTC. Similarly, a recent study has suggested that HYOU1 promoted cell growth and metastasis via activating PI3K/AKT signaling in epithelial ovarian cancer.^10^ Earlier evidence suggested that HYOU1 could promote angiogenesis via mediating secretion of VEGF.^20^, ^21^ However, molecular mechanism underlying oncogenic function of HYOU1 in cancer progression is not fully clarified.

HYOU1 is an ER‐resident chaperone. Its gene sequence contains a CCAAT motif‐rich cis‐element, identified as ER stress response element (ERSE),^22^ providing the structural basis for inducible expression in response to various ER stress stimuli such as hypoxia.^8^, ^23^, ^24^ Hypoxia, as a common feature in most solid tumors, can lead to activation of unfolded protein response in ER.^25^ Tumor cells could upregulate a class of pro‐survival molecular chaperones, protecting against ER stress‐induced cell death.^26^ Meanwhile, the hypoxia response system could upregulate glucose transporters and multiple enzymes of the glycolytic pathway to adapt to hypoxic environments through accentuated glycolysis.^27^ Increased glycolysis also provides glycolytic intermediates required for the biosynthesis of macromolecules and organelles of rapid tumor growth.^28^ As well as supporting needs of proliferation, cancer cells preferentially select aerobic glycolysis to facilitate invasion and metastasis regardless of ample oxygen availability.^29^, ^30^ Involvement of HYOU1 as a hypoxia response protein in reprogramming glucose metabolism is seldom studied. The present study demonstrated that silencing of HYOU1 markedly decreased glycolysis illustrated by less lactic acid production and glucose consumption.

Aerobic glycolysis is an emerging hallmark of cancer. Over two‐third of human cancers constitutively upregulate at least one glycolytic gene. Based on the inhibitory effect of HYOU1 on glycolysis, we observed the changes of expression for several glycolytic genes after silencing of HYOU1 in PTC cell lines. Our results confirmed that LDHB was significantly reduced when HYOU1 was silenced in IHH4 and K1 PTC cells. Furthermore, LDHB overexpression abrogated the suppressive effects of HYOU1 silencing on IHH4 and K1 PTC cells. Subsequently, we also confirmed that inhibition of glycolysis caused by HYOU1 silencing could be completely reversed by LDHB overexpression. These findings suggest that HYOU1 promotes glycolysis and aggressive progression of PTC through LDHB upregulation.

Lactate dehydrogenase (LDH) is among the enzymes mediating anaerobic glycolysis of tumor cells. LDH catalyses the bidirectional conversion of pyruvate and lactate. Active LDH is a homo‐ or hetero‐ tetramer molecule composed of two different subunits LDHA and LDHB, which are encoded by two distinct genes in human. LDHA is highly expressed in many types of cancers and considered as the key player of aerobic glycolysis.^31^ Unlike LDHA, the expression level of LDHB varies among different tumor cell types, and its role appears more complex. LDHB is silenced by promoter hypermethylation in some cancer types,^32^ while in others, it is highly expressed and involved in the aggressive progression of tumor.^33^, ^34^, ^35^, ^36^ It is generally thought that LDHA preferentially converts pyruvate to lactate, whereas LDHB preferentially converts lactate to pyruvate. However, recent evidence shows that both LDHA and LDHB could convert pyruvate to lactate.^37^ Furthermore, LDHB can compensate a majority of functions of LDHA under a condition of LDHA loss.^38^ Our results demonstrated that HYOU1 enhanced the stability of LDHB mRNA via its 3'UTR rather than synthesis of LDHB mRNA in PTC cell lines. It is well known that mature microRNAs can promote translational inhibition and/or mRNA decay by directly binding to complementary sequences located mainly in the 3'UTR of its target mRNAs.^39^ Our study supports that HYOU1 indirectly stabilizes LDHB mRNA by downregulating the expression of miR‐375 in PTC cell lines. We also found that the level of HYOU1 mRNA was significantly positively related to that of LDHB mRNA, while negatively associated with that of miR‐375 in PTC tissues. Furthermore, there is a significant inverse correlation between the level of HYOU1 mRNA and that of miR‐375 in PTC tissues. Cellular biogenesis of miRNAs is a multi‐step process and finely regulated at multiple levels. The exact mechanism underlying the regulation of miR‐375 by HYOU1 in PTCs remains to be further investigated in the future.

In conclusion, for the first time, our study provides evidence to support the involvement of HYOU1 in upregulating LDHB expression via indirectly downregulating the expression of miR‐375. We demonstrated that HYOU1 silencing suppressed LDHB expression, which was implicated in suppression of proliferation, migration and invasion of PTC through decreasing glycolysis. These findings may have important implications for understanding the molecular mechanisms underlying invasion and metastasis of PTC.

## CONFLICT OF INTEREST

The authors declared there are no competing financial interests.

## AUTHOR CONTRIBUTION

**Jia‐Mei Wang:** Investigation (equal); Methodology (equal); Writing‐original draft (equal). **Jingyi Jiang:** Writing‐original draft (equal); Writing‐review & editing (equal). **Da‐Lin Zhang:** Data curation (equal); Software (equal). **Xin Du:** Methodology (supporting). **Tong Wu:** Investigation (supporting). **Zhenxian Du:** Funding acquisition (lead); Supervision (lead); Writing‐review & editing (lead).

## Supporting information

Fig S1
