# Supplementary figures and images for: HYOU1 facilitates proliferation, invasion and glycolysis of papillary thyroid cancer via stabilizing LDHB mRNA
Source: J Cell Mol Med. 2021 Mar 31;25(10):4814–25. doi: 10.1111/jcmm.16453 (PMC8107106; doi:10.1111/jcmm.16453)

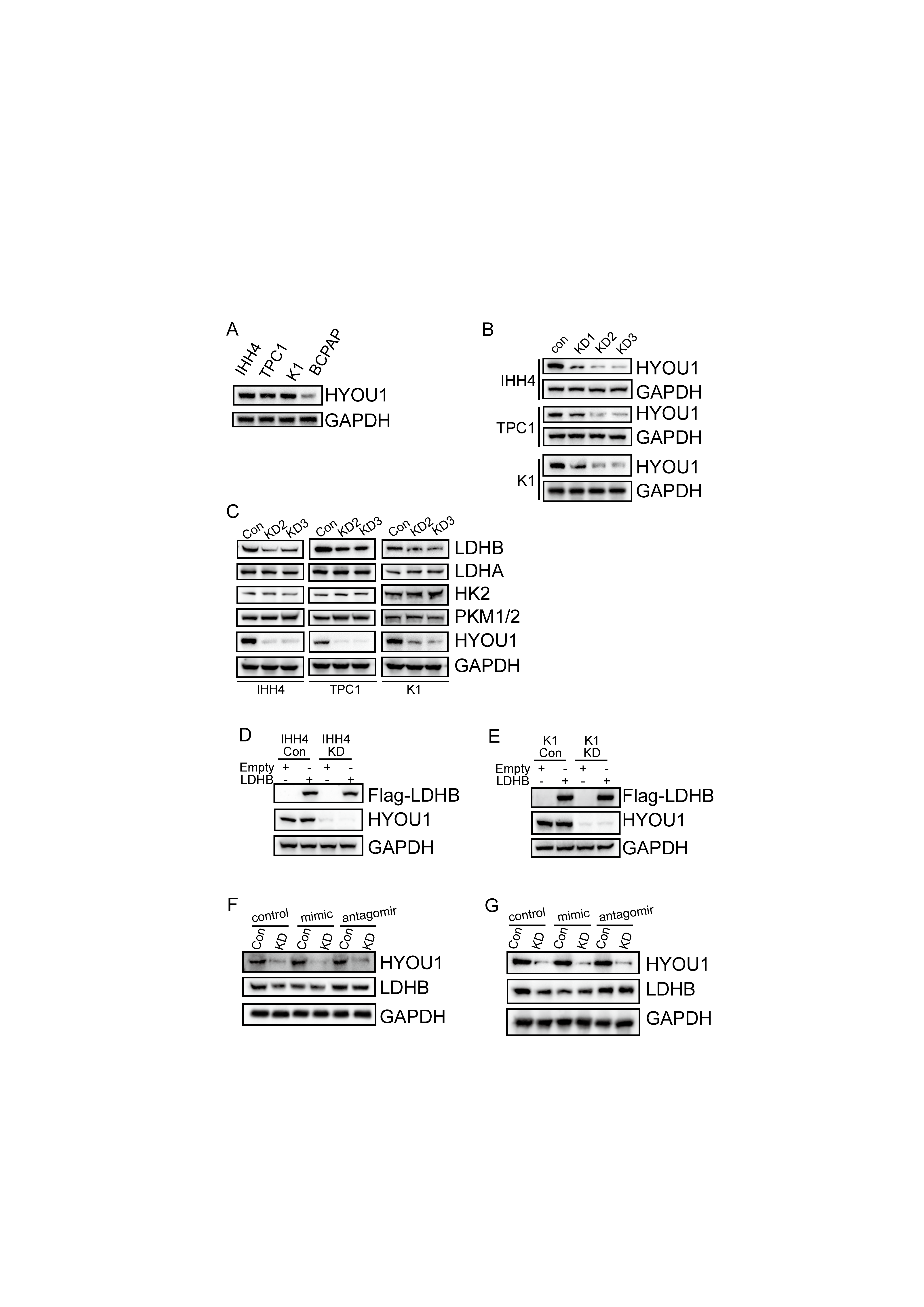

Supplement: Supplementary file 1 — Fig S1 [file JCMM-25-4814-s001.tif]
